# Appendiceal stump closure by metal endoclip in the management of complicated acute appendicitis

**DOI:** 10.1186/1749-7922-8-35

**Published:** 2013-09-18

**Authors:** Carlos Augusto Gomes, Cleber Soares Junior, Rodrigo Oliveira de Peixoto, Jose Murillo Bastos Netto, Camila Couto Gomes, Felipe Couto Gomes

**Affiliations:** 1 Department of Surgery, Hospital Universitário (HU), Universidade Federal de Juiz de Fora (UFJF) - Faculdade de Ciências Médicas e da Saúde de Juiz de Fora (SUPREMA) -Brasil, Bairro Bom Pastor, Minas Gerais, Brasil; 2 Department of Surgery, Hospital Universitário (HU), Universidade Federal de Juiz de Fora (UFJF), Juiz de Fora, Brazil; 3 Internal Medicine Departament, Hospital Universitário (HU), Universidade Federal de Juiz de Fora (UFJF), Juiz de Fora, Brazil; 4 Morphology Department, Faculdade de Ciências Médicas e da Saúde de Juiz de Fora (SUPREMA), Juiz de Fora, Brazil

**Keywords:** Complicated appendicitis, Appendectomy, Laparoscopy, Appendiceal stump

## Abstract

**Background:**

Closure of appendicular stump has been performed in different ways; however, the use of the metal endoclip in complicated grades of acute appendicitis, has not been evaluated yet in a prospective way.

**Objective:**

To establish the effectiveness of appendiceal stump closure by metal endoclip for complicated appendicitis.

**Method:**

From January 2009 to January 2011 were evaluated 131 consecutive patients who underwent a laparoscopic appendectomy for complicated acute appendicitis. From those, 118 underwent appendiceal stump closure by metal endoclip. The patient’s age ranged from 12 to 75 years old (31.7 ± 13.3) and 52.7% were male. Complicated appendicitis refers to gangrenous and/or perforated appendix, which may lead to abscess formation and degrees of peritonitis. The outcomes viability, operative time, infection complication, operative complications, and conversion rate were chosen to evaluate the procedure.

**Results:**

The appendiceal stump closure by metal endoclip was used in 90% of cases. The presence of appendix base necrosis was the most important factor involved in failure of the procedure. Laparoscopic knot (1.5%), laparoscopic endo-suture (3.8%) and video assisted laparotomy (4.7%) were the alternatives used in difficult cases. The mean operative time was (67.54 ± 28.13 minutes). The wound and intra-abdominal infection rates were 2.54% and 5.08%, respectively. There were no operative complications and the conversion rate was 0.85%.

**Conclusion:**

The appendiceal stump closure by metal endoclip, in complicated grades of acute appendicitis, is a safe and effective procedure. In patients with appendix base necrosis it should be avoided in favor of other alternatives.

## Introduction

Appendicular stump closure is an important step during appendectomy as its inappropriate management can lead to serious post-operative complications. The development of life-threatening problems such as stercoral fistulas, postoperative peritonitis, and sepsis is feared and unwanted. In 1985, Engstrom and Fenyo [1] showed that the tactical modification of appendiceal stump closure, with a single endoligature replacing the invaginating suture, is a safe and effective approach. The modification adjusted very well with demonstrated benefits such as simplicity and decreased operation time and anatomic deformities at the cecum. Nowadays, it has become the procedure of choice during a laparoscopic appendectomy [1].

Appendiceal stump has been secured by different ways during laparoscopic appendectomy, including the use of mechanical endo-stapler [2-4], endo ligature (endo-loop®) [5-7], metal endoclips [8-11], bipolar endocoagulation [12], polymeric endoclips [13,14], and intracorporeal suture [15]. All these alternatives have advantages and disadvantages during the different clinical stages of the acute appendicitis, and none of them have been assessed by any prospective randomized (and experimental) studies [2].

In addition, the appendiceal stump treatment by metal endoclips in complicated acute appendicitis has not been evaluated yet by any prospective clinical research. Therefore, the objective of this study was to verify the safety and effectiveness of appendicular stump closure by metal endoclips, in complicated stages of acute appendicitis.

## Method

From January 2009 to January 2011 were evaluated 131 consecutive patients who underwent a laparoscopic appendectomy for complicated acute appendicitis. From those, 118 underwent appendiceal stump closure by metal endoclip. The patient’s age ranged from 12 to 75 years old (31.7 ± 13.3) and 52.7% were male.

The study was approved by the Ethics and Research Commission at the Monte Sinai Hospital in Juiz de Fora – Minas Gerais – Brazil, and all patients signed up an informed consent term. Three senior surgeons who are titled by the Brazilian Society of Laparoscopic Surgery participated in it.

Complicated appendicitis refers to gangrenous and/or perforated appendix, which may lead to abscess formation and degrees of peritonitis. The laparoscopic grading system of acute appendicitis was used to graduate the disease (Table 1) [16]. All patients were operated under general anesthesia and received 2 g amoxacillin-clavulanate intravenously in the perioperative period. The patients with complicated disease were then treated with double antibiotic coverage of metronidazole (1.5 g/day) and ceftriaxone (2 g/day – patients who are allergic to penicillin received ciprofloxacin 400 mg/twice daily) until white blood cell count reached normal limits and temperature was lower than 38º C. The recommended mean time of antibiotic coverage ranged from 5 to 10 days according to grade and clinical course.

**Table 1:** Laparoscopic grading system of acute appendicitis according to macroscopic inflamatory findings

**Grade**	**Laparoscopic findings**
Grau 0	Normal looking appendix
Grau 1	Hiperemia e oedema of appendix
Grau 2	Fibrin
Grau 3 A	Segmentar necrosis
Grau 3 B	Appendicular base necrosis
Grau 4 A	Abscess
Grau 4 B	Regional peritonitis
Grau 5	Diffuse peritonitis

From Gomes et al, 2012 [16].

**Figure 1:**
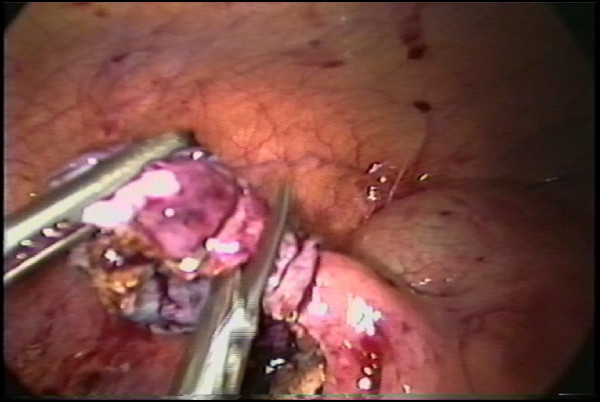
Appendiceal stump closure by metal endoclip in complicated acute appendicitis grade 3A.

The operation was performed by three ports and they were located in the umbilicus to introduce a 30 ^0^ Karl Storz® (23006 BA) optic, in the suprapubic midline (5 mm), and in the left lower quadrant (12 mm). Then, the patient was positioned in the Trendelenburg with a mild left tilt, to facilitate the exposure of the right lower quadrant. The appendiceal stump closure was performed by applying two T 400 metal endoclips (Ethicon Endo-Surgery^®^), in a health tissue next to the cecum wall (distance of less than 3.0 mm) and other one around the appendix base. After the appendix section, the extraverted appendiceal mucosa was endocoagulated (Figure 1). Laparoscopic knot, laparoscopic endo-suture and video assisted laparotomy [17] were the alternatives used in difficult cases. The abdominal cavity was judiciously irrigated with warm saline solution and suctioned dry under direct visualization. Then, the appendixes were removed from the abdominal cavity in a retrieval bag and sent for histopathological study.

Operative time that lasts from skin incision to skin suture was measured in terms of minutes. Surgical site infection was defined by clinical signs of edema, redness around the wound, or purulent discharge until the 30th postoperative day. The diagnosis of intra-abdominal infections was suspected by clinical signs and demonstrated by ecography, computed tomography, or laparoscopy. Operative complications were defined as bleeding, iatrogenic injury, small bowel obstruction, endoclip escape, or enteric leak.

The viability of appendiceal stump secured by metal endoclips, operative time, infection complication, operative complication, and conversion rate were the outcomes chosen to study the effectiveness of the procedure. *Statistical Package for Social Sciences* (SPSS) 13.0 version for *Windows* was used as data bases.

## Results

### Viability of appendiceal stump closure by metal endoclips according to laparoscopic grading system of acute appendicitis (n = 131)

The appendiceal stump closure by metal endoclip was viable in 118 (90%) patients who underwent laparoscopic appendectomy for complicated grades of acute appendicitis. In others one considered difficult cases, were used alternatives which the frequencies can be found in Table 2. It must be taken in account that the presence of appendix base necrosis was the most important factor involved in failure of the procedure (Table 2).

**Table 2:** Alternatives of appendiceal stump closure in complicated grades of acute appendicitis (n = 131)

**Apendiceal stump closure**	**Laparoscopic grading system**					**Total**	
**Alternatives**	**Grade 3A**	**Grade 3B**	**Grade 4A**	**Grade 4B**	**Grade 5**	**n**	**%**
Clip	45	3	31	21	18	118	90
Laparoscopic knot	0	0	1	1	0	2	1.5
Laparoscopic suture	0	4	0	1	0	5	3.8
Laparotomy	0	5	1	0	0	6	4.7
( n ) *	45	12	33	23	18	131	100
(%) ^#^	(34.4)	(9.2)	(25.2)	(17.6)	(13.7)	100	(100)

Note: *Absolute number by specified grade. ^#^Relative number by specified grade.

### Mean operating time spent during laparoscopic appendectomy for complicated grades of acute appendicitis compared with four other similar studies (n = 118)

The mean operative time spent during laparoscopic appendectomy for complicated grades of acute appendicitis was 67.54 ± 28.13 minutes. Patients with abscess presented the highest operative time (78.24 ± 28.72 minutes). This time was then compared with four other similar studies (Table 3).

**Table 3:** Mean operating time spent during laparoscopic appendectomy for complicated grades of acute appendicitis from four similar studies (n = 118)

**Mean operating time in complicated laparoscopic appendectomy**			
**Study or subgroup**	Mean*	**SD**^**#**^	**n**^**§**^
Katsuno et al., 2009 ( 17 )	116.7	45	141
Lin et al., 2006 ( 18 )	96.1	43.1	99
So et al., 2002 ( 19 )	73	25	85
Khalili et al., 1999 ( 20 )	86	29	122
^μ^ Gomes et al., 2012	67.4	28.1	131

Note:* Mean operating time in minutes. ^#^ Standard deviation.^§^ Sample of each series. ^μ^ (Author’s series).

### Conversion and postoperative infection rates, among patients operated on for complicated grades of acute appendicitis which the appendiceal stump was closured by metal endoclip (n = 118)

The conversion rate in this series of 118 patients occurred in only one (0.85%) with abscess. In nine out 12 (84%) patients with appendiceal base necrosis the appendiceal stump closure by metal endoclip was not possible. Moreover, there were no complications defined as operative complications or related to the metal endoclip. Surgical site infections were diagnosed as wound infection in three patients (2.54%) and as intra-abdominal in six patients (5.08%) and the patients altogether had uneventful recovery (Table 4).

**Table 4:** Conversion and postoperative infection rates among patients operated on with complicated appendicitis which the appendiceal stump was closured by metal endoclip (n = 118)

**Parameter**	**Event**^**#**^	%*
Conversion rate	1	0.85%
Wound infection	3	2.54%
Intra-abdominal infection	6	5.08%
Postoperative complications	0	0

Note: *(Relative number). ^#^Event (Absolute number).

## Discussion

The treatment of appendiceal stump by metal endoclip was evaluated with the help of four clinical studies [8-11]. Two of these prospectives studied the treatment’s effectiveness in non complicated forms of appendicitis, and despite the small size of sample used; they recommended its use [8,10]. Another study enrolled 233 patients retrospectively, from 2005 to 2010 [11]; however, its methodology failed to include non complicated and complicated forms of the disease. Moreover, it omitted the percentages among them and did not comment on any grading system. Thus, the comparison of the results of this research is hampered. The study of Gomes & Nunes [9] included non complicated and complicated forms of acute appendicitis, like the previous one; however, the survey was not designed with this objective and, therefore, the results are observable.

Thus, this series stands out from the others, as it evaluated the effectiveness of the procedure only in complicated grades of acute appendicitis. The research has shown that the treatment of appendiceal stump by metal endoclip was feasible in 90% of cases. In nine out 12 (84%) patients, the presence of appendiceal base necrosis (grade 3B), represented the most important aspect of procedure failure and other alternatives were required. In remaining three patients, the endoclip could be positioned in a healthy segment of the cecum wall. Therefore, the presence of local or diffuse peritonitis was not a disincentive event for the procedure (Tabela-1).

The mean operative time of 67.4 ± 28.1 minutes was the lowest when compared with the four other studies [18-21], but is similar to that reported by So et al. [20] in which all patients were operated by trained surgeons. The patients with abscess had longer operative time, which may explain the presence of more advanced cecum-appendiceal inflammatory processes and more a difficulty operation (Table 3).

Infection complications (wound and intra-abdominal) are two parameters traditionally used to validate the safe and effectiveness of laparoscopy in the management of acute appendicitis [3]. Similarly, we can evaluate the appendiceal stump closure with metal endoclips with them. A point that should be highlighted is that the diagnosis of surgical site infection would more accurately require positive organism culture confirmation rather than just a clinical diagnosis, as used in this series and in others. A meta-analysis that compiled 11 studies has shown that from the 2175 operated patients with complicated acute appendicitis, 92 (4.2%) had infection related to the wound [22]. Therefore, the frequency of 2.54%, observed in this series, was low and is in agreement with the literature (Table 4).

The frequency of intra-abdominal infection in the same study was 5.9% (1059 patients/63 events) [22]. The copious irrigation of the abdominal cavity with saline solution 0.9% is cited as a possible cause of its development, proposing a judicious local irrigation, accompanied by aspiration and the application of gauze [23]. Katkhouda et al. [24] have reduced its frequency from 2.4% to 0.4%, with the implementation of a laparoscopic surgery service and some simple per-operative care such as exposure of the appendicular base; concern with fragments, gaps and the appendicolith; inspection, irrigation and aspiration of the bottom of the peritoneal cavity; and the use of endobags. The frequency of intra-abdominal infection of 5.08% was similar and moreover, of the six patients with intra-abdominal collection, four of them were treated exclusively with antibiotics and the other two by means of percutaneous drainage guided by abdominal ultrasound (Table 4). No patient required reoperation, and all of them had uneventful recovery. In this context, the treatment of the appendiceal stump using metal clips qualifies as a safe and effective alternative.

The need of laparotomy is variable in its frequency and can reach 10 to 39.7% [25,26]. Among the assigned factors are adhesions, localized perforation, diffuse peritonitis, appendix base necrosis, retrocecal position, bleeding, and inability to identify the organ, appendicular tumor, and iatrogenic lesions [27]. In this series of 118 studied patients, laparotomy was necessary in only one (0.85%) with abscess. This low conversion rate can be explained, because in nine out 12 (84%) patients with appendiceal base necrosis, the management of appendiceal stump by metal endoclip was not possible and they could not be included in the analysis. Anyway, the appendiceal base necrosis was the most important factor responsible for procedure failure during treatment of complicated appendicitis. The use of mechanical stapler can circumvent the problem; however, because of its cost, it was not used in this series.

There were no complications related to the endoclip during its release or the fragmentation of cecum-appendicular tissue when it is placed during operation. The fact of not performing its twisting can, at least partly, explain the difference with another study published recently [11].

Therefore, despite being a nonrandomized study without considering the body mass index and the cost of the procedure, it draws attention to an approach, independent of the diagnosis of complicated or non-complicated acute appendicitis. It is the choice of treatment of appendiceal stump in our Service and not just mere philosophical question. Moreover, it reserves the use of endo stapler for patients with disease located in appenciceal base and appendix with large diameter. In conclusion, the appendiceal stump closure by metal endoclips, in complicated grades of acute appendicitis, is a safe and effective procedure. In patients with necrosis and large appendiceal base, the use of metal endoclips should be avoided in favor of other alternatives.

## Competing interests

Drs. Carlos Augusto Gomes, Cleber Soares Junior, Rodrigo de Oliveira Peixoto, José Murilo Bastos Netto, Camila Couto Gomes and Felipe Couto Gomes have no conflicts of interest or financial ties to disclose.

## Authors’ contributions

CAG: conception and design, acquisition of data, analysis and interpretation of data, entire manuscript reviewer. CSJ and RPO: acquisition of data and revising it critically for importante intellectual content; JMBN: analysis and interpretation of data and revising it critically for important intellectual content; CCG and FCG: have made substantial contributions to data collection, graphic art and contribution in the revision process. All authors read and approved the final manuscript.

## Authors’ information

Carlos Augusto Gomes: MD. PhD. Associate Professor, Surgery Department, Hospital Universitário (HU), Universidade Federal de Juiz de Fora (UFJF) and Faculdade de Ciências Médicas e da Saúde de Juiz de Fora (SUPREMA) – Brasil. TCBC (Membro Titular do Colégio Brasileiro de Cirurgiões)
